# Co-management as a Catalyst: Pathways to Post-colonial Forestry in the Klamath Basin, California

**DOI:** 10.1007/s10745-016-9851-8

**Published:** 2016-10-07

**Authors:** Sibyl Diver

**Affiliations:** 1 Department of Environmental Science, Policy, and Management, University of California - Berkeley, Berkeley, CA USA; 2 Department of Earth System Science, Stanford University, Y2E2 Building, 473 Via Ortega, Room 140, Stanford, CA 94305-4216 USA

**Keywords:** Co-management, Forest policy, Indigenous knowledge, Land management, Environmental governance, Cultural and ecological restoration, Karuk Tribe, Klamath River, Self-determination, Prescribed burning

## Abstract

Co-management frameworks are intended to facilitate sustainable resource management and more equitable power sharing between state agencies and Indigenous communities. However, there is significant debate about who benefits from co-management in practice. This article addresses two competing perspectives in the literature, which alternately portrays co-management as an instrument for co-optation or for transformation. Through a case study of co-management negotiations involving the Karuk Tribe and the U.S. Forest Service in the Klamath Basin of Northern California, this study examines how Indigenous communities use co-management to build greater equity in environmental decision-making, despite its limitations. The concept of pivot points is developed to describe how Indigenous communities like the Karuk Tribe are simultaneously following existing state policies and subverting them to shift federal forest management. The pivot point analytic demonstrates one mechanism by which communities are addressing Indigenous self-determination goals and colonial legacies through environmental policy and management.

“We want to manage the forest traditionally. In traditional management, fire is the primary tool, so we need to get fire [back] on the landscape. And because of our legal situation, we need to do it with the Forest Service. We need to be able to do it in a co-management capacity, to be able to manage public trust and tribal trust resources simultaneously.”

– Ron Reed, Karuk tribal member

## Introduction

Co-management refers to the sharing of management power and responsibility between government agencies and local people, typically through a formal agreement (Berkes *et al*. 1991; Berkes and Turner 2006). The co-management concept has been developed through common pool resources scholarship, which questions the ability of centralized bureaucracies and deregulated markets to respond to highly contextualized environmental management problems and instead emphasizes the importance of including local knowledge and interests in environmental decision-making (Jentoft *et al*. 1998; Ostrom 1990). Although co-management does not apply exclusively to Indigenous communities, the co-management term was initially used in court decisions providing U.S. treaty fishing tribes with the right to “concurrent management,” and has since evolved to mean “cooperative” or “collaborative” management (Pinkerton 2003).

The literature alternately portrays co-management as a worthwhile endeavor that has the potential to transform natural resource conflicts to achieve more sustainable management (e.g., Pinkerton 1989), and as a state-driven project that co-opts community interests (e.g., Nadasdy 2003). With cooperative management, there are always concerns of community or agency interests being captured by the other party (Singleton 2000). Risks of co-optation are a particular challenge for Indigenous communities working to achieve greater self-determination, a term that signifies the ability of Indigenous communities to participate meaningfully in the creation of the government institutions that they live with (Anaya 1993). This is, in part, because Indigenous relationships with state-based resource management institutions are embedded within colonial systems that have historically excluded Indigenous communities from land management decisions (e.g., Taiepa *et al*. 1997).

These issues have informed a key debate in the co-management literature over the extent to which co-management arrangements can facilitate more equitable power sharing in practice. Through an in-depth case study of co-management between the Karuk Tribe and the U.S. Forest Service in the Klamath Basin, Northern California, USA, this article engages with competing views regarding co-management effectiveness, and addresses a gap in our understanding of how co-management processes can evolve to build greater equity in natural resource management. This work is particularly concerned with understanding how Indigenous communities are strategically responding to state-based institutions, given existing power asymmetries. My analysis will show how ongoing tensions between co-optation and transformation contribute to the co-management process. I draw on these tensions to develop the concept of *pivot points*, which refers to existing government policies that provide a starting point for Indigenous communities to negotiate meaningful policy change.

### Co-management Binaries: Transformation or Co-optation?

Scholars have emphasized the potential for co-management to shift norms and transform environmental policy (Carlsson and Berkes 2005). Two mechanisms by which policy transformations take place include incorporating local knowledge and facilitating social learning within environmental governance processes. By accounting for community knowledge and interests, co-management has been found to produce highly desirable environmental outcomes such as reduced harvest pressure and increased regulatory compliance, alongside benefits for local livelihoods (Cinner *et al*. 2012; Jentoft 2005). By facilitating social learning, where learning involves “becoming a full participant in a socio-cultural practice” (Lave and Wenger 1991:29), co-management processes can help “transform social relations and generate less conflictual ways of addressing difficult joint problems” (Pinkerton 2003:70). As one well-known example, Pacific Northwest fisheries co-management is recognized for improving regional understanding of fisheries dynamics—in part by creating new monitoring systems and coordinating decision-making among nested institutions (Diver 2012; Pinkerton 1989, 1992).

Co-management supporters recognize that not all co-management efforts are successful, or the same (Agrawal 2000; Armitage *et al*. 2007; Borrini-Feyerabend *et al*. 2004). To better understand what constitutes effective co-management, researchers have analyzed the conditions under which co-managers have achieved their goals, e.g., by improving resource sustainability or reducing social conflict (Armitage *et al*. 2009; Pomeroy and Berkes 1997). Scholars have also examined conditions favoring “complete co-management” that achieve more equitable power-sharing (Pinkerton 2003) or “adaptive” co-management that encourage evaluation and learning (Olsson *et al*. 2004). In addition, co-management arrangements are often described on a continuum, where the degree of community participation ranges from more consultative to more community-driven arrangements (Berkes 1994). This approach builds on Arnstein’s (1969) critique of lower level participatory arrangements, where community participation promotes the legitimization of status quo inequities.

Visualizing a “ladder of participation” for co-management (Arnstein 1969; Berkes *et al*. 1991) leads to the question of how often joint decision-making arrangements reach the highest rung. Due to the persistence of uneven power relations between so-called “co-managers,” some researchers have viewed co-management as a tool for the co-optation of Indigenous interests (Feit and Spaeder 2005; Nadasdy 2003). Despite some successful initiatives, many co-management initiatives fail to facilitate meaningful power-sharing, in part because bureaucratic structures privilege state positions and dominant knowledge systems often exclude Indigenous worldviews (Deloria and Lytle 1984; Nadasdy 2007; Natcher *et al*. 2005; Spak 2005; Usher 2000; Weir 2009). In addition, agreements are not always enforced because this requires sufficient levels of legal accountability, funding support, enforcement personnel, and dispute resolution capacity—all of which can be difficult to achieve (Diver 2012; Mabee and Hoberg 2006). Thus, there is a risk of co-management becoming a tool for “legitimizing existing practices” (Trosper *et al*. 2012:184) and excluding Indigenous community interests.

Sharing Indigenous knowledge with agencies through co-management can also be highly problematic. Indigenous knowledge systems often have distinct qualities that diverge from Western scientific standards and dominant policy frameworks (e.g., Barnhardt and Kawagley 2005). For example, government agencies are not typically structured to accommodate Indigenous ontologies or epistemologies that emphasize spiritual relationships between people and the landscape. Translating Indigenous management concepts into narrow categories that fit within pre-defined agency structures often results in incomplete representations of complex Indigenous knowledge systems (Vaughan 2012; Weir 2012). Furthermore, when knowledge systems diverge, dominant institutions typically choose Western science as the final authority (Nadasdy 2003). Another problem occurs when bureaucracies limit definitions of Indigenous knowledge to the category of pre-contact “traditional” practices as a strategy for limiting Indigenous claims to land and resources (Vermeylen 2013).

Co-management with Indigenous communities also raises the issue of knowledge capture, where giving away knowledge diminishes community control over sensitive natural resources (Weinstein 1998). This is especially problematic when hunting, fishing, or gathering occurs under open-access conditions. As an additional concern, government agencies that have an inventory of cultural information on file may be less likely to engage directly with Indigenous community representatives, which works against the principles of Indigenous self-determination and meaningful consultation.

To address the tensions between transformation and co-optation, co-management researchers have described different models of how state agencies interact with Indigenous communities. Smith (2013:93) contrasts “coexistence” models that account for Indigenous interests to “assimilationist” models that follow the principle, “you cooperate, we’ll manage.” Other studies suggest that agency-community relations may shift through the co-management process and yield unexpected changes in power-sharing over time, as with Pacific Northwest treaty fisheries (Diver 2012; Singleton 1998). In some cases, Indigenous communities have leveraged co-management to achieve incremental gains, such as creating new Indigenous management institutions that facilitate broader community participation in decision-making (Natcher 2000). Or co-management may shift *de facto* rights (rights in practice) that enhance community control, even in the absence of *de jure* or legal rights (Galappaththi and Berkes 2015).

Such examples illustrate a politically savvy approach, where Indigenous communities leverage collaborative management forums as a strategic advocacy platform for self-determination, while recognizing the limitations of such forums (e.g., Willow 2015). Bruyneel’s (2007) concept of the “third space of sovereignty” furthers this idea by exploring how U.S. Indigenous communities have simultaneously worked inside and outside existing government structures to transcend zero sum gain conflicts over territory. This perspective recognizes that Indigenous-led institutions are always operating within imposed political constraints, but also asserts that Indigenous peoples choose for themselves how and when to operate within these constraints (Bruyneel 2007; Cornell 2013).

The pivot point idea developed in this article extends Bruyneel’s (2007) “third space of sovereignty” concept to the realm of environmental management. My case analysis discusses how Indigenous communities like the Karuk Tribe are simultaneously following existing state policies and subverting them, despite the tendency of state-based institutions to exclude Indigenous worldviews. This approach acknowledges the ongoing influence of colonial histories, but also demonstrates a pathway towards greater power sharing—by locating strategic pivot points within existing environmental regulations and planning processes. After presenting the case background, I develop the pivot point concept by analyzing three aspects of Karuk-Forest Service negotiations over mid-Klamath forest restoration: 1) crisis moments motivating co-management, 2) transformational moments in the co-management process, and 3) structural barriers to equity in Indigenous-state co-management arrangements.

## Case Background: Co-management Negotiations Between the Karuk Tribe and the U.S. Forest Service

This case study addresses forest management negotiations between the Karuk Tribe and the U.S. Forest Service that culminated in a *de facto* co-management initiative, the Ti Bar Demonstration Project (pronounced TEE Bar). Although the U.S. Forest Service has not adopted the term “co-management” as official policy (Mitchell 1997:58), the agency regularly enters into contracts and collaborative agreements to facilitate joint projects with federally recognized tribes. This case was selected because of the Karuk Tribe’s combined commitment to Indigenous self-determination and environmental sustainability, both important elements of co-management. This case is also important because of the Karuk Tribe’s persistence in negotiating to increase access to their ancestral territory, regardless of having limited organizational capacity and legal leverage.

**Fig. 1:**
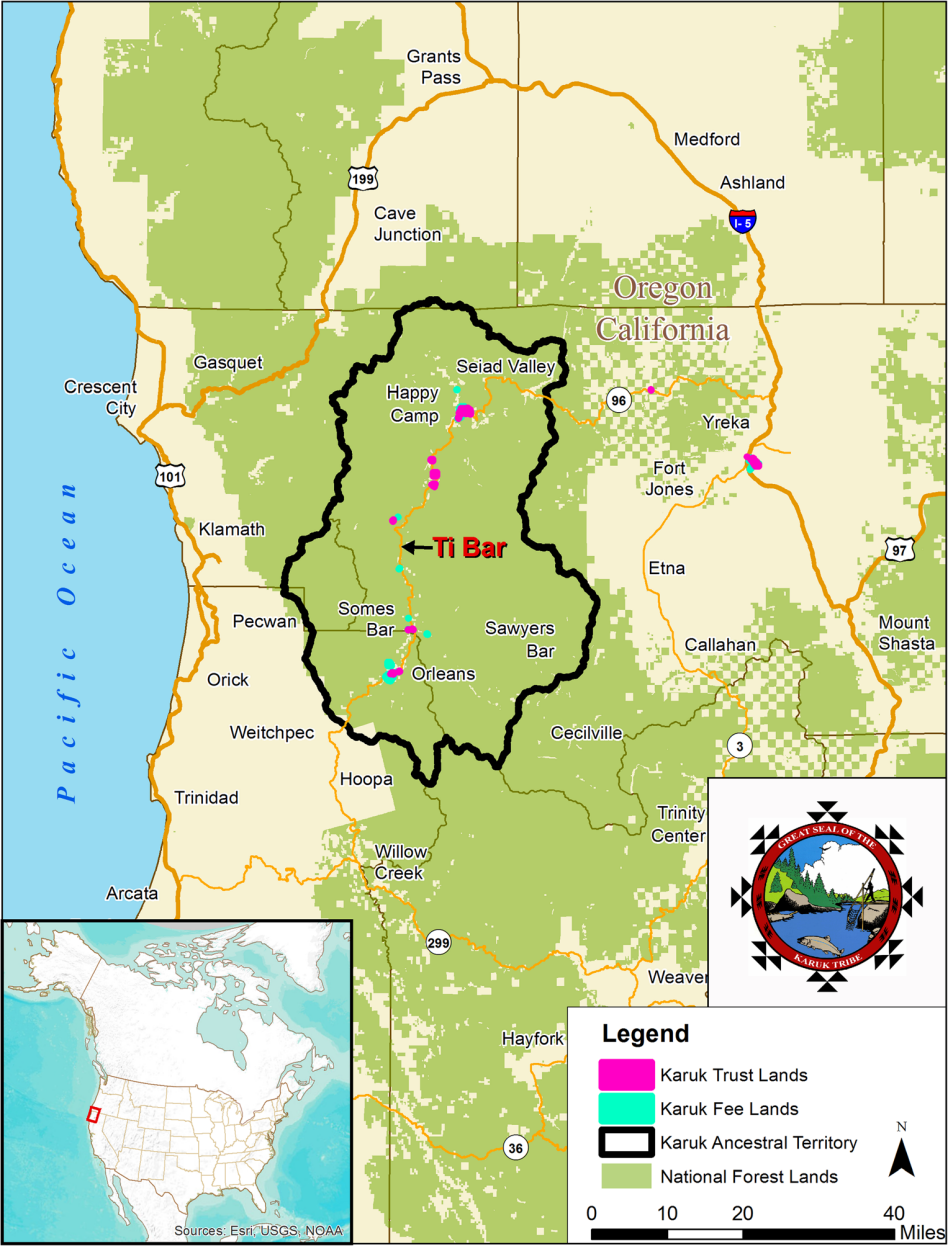
Map of Karuk Aboriginal Territory (California and Oregon, U.S.) with overlapping National Forest areas. The Ti Bar Demonstration Project site is located approximately halfway between the towns of Orleans and Happy Camp. Map by Jill Beckmann, Karuk Tribe Department of Natural Resources

The Karuk people come from the middle section of the Klamath River, an ecologically diverse and mountainous area near the border between California and Oregon. Karuk ancestral territory covers approximately 1.38 million acres (Fig. 1). Despite a history of displacement, many Karuk tribal members have maintained a strong connection to their homelands (Karuk DNR 1995; Salter 2003). Tribal members continue to harvest subsistence foods from the forest and river, including salmon (Reed and Norgaard 2010). Karuk people have also maintained a longstanding tradition of gathering at established cultural sites to practice World Renewal ceremonies. Karuk World Renewal philosophy obligates its followers to take on stewardship responsibility for natural resources, an important mandate for tribal land managers (Karuk DNR 2011; Kroeber and Gifford 1949; Lake *et al.* 2010).

The Karuk Tribe is federally recognized by the U.S. government but in contrast to neighboring recognized tribes, the Karuk do not have a reservation. A small number of tribal trust lands are scattered throughout the territory, with parcels adding up to just over one square mile in total area (personal communication, Scott Quinn, May 28, 2014). This is because of California state history, which has provided limited opportunities for the recognition of Indigenous homelands. Although the Karuk Tribe historically negotiated treaties they were not ratified by the U.S. Senate due, in part, to the massive land grab occurring during the California Gold Rush (Heizer 1972; Hurtado 1988; Johnston-Dodds 2002). In 1905, the U.S. federal government designated most of Karuk territory as Forest Reserves, now National Forests (Bower 1978). Because no valid treaty was signed to legally cede Karuk territory to the U.S. government, the Karuk Tribe continues to dispute the legitimacy of federal ownership over its territory (Karuk DNR 2011).

As one ongoing conflict in Karuk-Forest Service relations, Karuk land management systems that include prescribed burning were largely displaced from the mid-Klamath region. This is due to massive social, political, and environmental changes occurring in the mid-1800s and onwards (Bright 1978; Huntsinger and McCaffrey 1995; Norton 1979). For many California tribes, prescribed burning, or the intentional human use of low intensity fire, has been an essential land management tool allowing communities to check forest succession and enhance desired understory vegetation (Anderson 2005, 2006). Karuk people have long used fire to manage Klamath forests as a mosaic of habitats, which promote a diversity of cultural resources, including the plant and animal species used for Karuk subsistence foods and ceremonial regalia (Busam 2006; Lake 2007; Salter 2004). In contrast, U.S. forest management has been dominated by policies supporting fire suppression, which have largely prevented the use of prescribed burning (Biswell 1989; Pyne 1982; Timbrook *et al*. 1982). Many Karuk people directly link U.S. fire suppression policy to the decreased production of Karuk cultural resources that depend on fire disturbance, such as basket-weaving plants, acorn-producing oak trees, and wildlife species like deer and elk (Norgaard 2014; Salter 2004).

Following a resurgence of tribal self-governance institutions in the 1970s and 1980s, Karuk leaders have advocated for increased tribal management authority over federal forestlands that overlap with Karuk territory, especially areas used for ceremonies and subsistence purposes (Diver *et al*. 2010). Karuk-Forest Service negotiations have been characterized by extreme conflict. This includes disputes over protecting sacred sites from logging such as the G-O Road case (Grieser *et al*. 2008), ongoing struggles to use prescribed burning for enhancing cultural resources within federal forests (Busam 2006; Lake 2007), and problems with maintaining tribal access to harvesting areas (Karuk DNR 2011; Norgaard 2005).

In its negotiations with the Forest Service, the Karuk Tribe has leveraged the concept of federal trust responsibility, which directs the U.S. federal government to uphold a fiduciary duty to manage trust resources for the benefit of tribes as distinct political bodies (Wilkinson and AILTP 2004:51-62). Forest Service policies recognize the special status of U.S. tribes as having governments and laws that preexisted the U.S. Constitution (Mitchell 1997:33). Thus, federally recognized tribes like the Karuk are legally authorized to hold special use rights within federal forests. For example, the Forest Service has confirmed the rights of tribal members to gather non-timber forest projects for non-commercial use on federal lands without a permit through its Pacific Southwest Region Traditional Gathering Policy. With the Tribal Forest Protection Act of 2004, U.S. federal law also supports tribal management activities in federal forests when there are threats to adjacent tribal trust lands. Furthermore, the Indian Self-Determination and Educational Assistance Act of 1975 directs the U.S. federal government to assure the maximum participation of Indigenous people in programs affecting Indigenous communities, and to support tribes in developing strong tribal governments.

### Ti Bar Demonstration Project

In the mid-late 1990s, the Karuk Tribe and the Forest Service initiated the Ti Bar Demonstration Project (Ti Bar Demo) as a key moment in their negotiations regarding the co-management of priority Karuk cultural areas within the Klamath National Forest. Ti Bar refers to a river bar and the surrounding sub-watershed where Ti Creek flows into the Klamath River near Somes Bar, CA. Karuk tribal land managers view Ti Bar Demo as one of their most successful collaborations with the agency to date. Authorized by an Interagency Agreement between the U.S. Forest Service and Bureau of Indian Affairs (BIA), the project aimed to 1) demonstrate “culturally appropriate” management techniques and 2) develop effective processes to “jointly undertake projects.” Importantly, Ti Bar Demo was not a pre-formed project; rather, Karuk tribal land managers were project co-leads and proposed priority restoration treatments. Through Ti Bar Demo, Karuk managers applied a new eco-cultural restoration approach to land management that included prescribed burning—a significant departure from earlier production forestry approaches. The project was partially completed, and then abandoned around 2000 following a Forest Service leadership change.

## Methods

I conducted this case study over a five-year period from fall 2009 to spring 2014. Methods included semi-structured interviews with key informants, participant observation, and document analysis. Interviews followed the snowball sampling method, a technique in which existing study participants recruit additional respondents from among their acquaintances (Goodman 1961). I selected 30 key informants (10 Karuk Tribe staff, 14 Forest Service staff, and 6 additional community members) who played a role in the Ti Bar Demonstration project or related initiatives.

Participant observations varied over the study period from visiting for several weeks during intensive research periods, to attending monthly events, to joining regular conference calls. I attended Karuk Department of Natural Resources (Karuk DNR) planning sessions, walked through Ti Bar Demo management and restoration sites with project participants, joined Karuk-Forest Service field trips, observed Karuk ceremonies and subsistence activities, assisted with youth workshops on traditional foods revitalization, and witnessed community wildfire responses. I also attended Forest Service listening sessions on sacred site policies, conferences on prescribed fire, and mid-Klamath community-Forest Service planning sessions.

This project followed a community-engaged scholarship approach (see Diver 2014; Diver and Higgins 2014). I developed research questions with community partners at the Karuk DNR and reviewed work in progress with local mentors. I worked through existing tribal approval processes, and received approval from the Karuk Tribal Council for my research proposal in May 2012. I also worked with tribal managers to develop a new research protocol governing respectful collaborations with the Karuk Tribe. Alongside my research, I helped develop the Karuk-UC Berkeley Collaborative, an organization that builds connections between tribal members and the UC Berkeley community to support Karuk eco-cultural revitalization.

## Results

### Getting to Co-management: Crisis Moments and Resisting Co-optation

“Our policies were very important to the Karuk, because they didn’t have land. So that particularly added to difficulties and tension. Where tribes have land, they do their thing. . . . I think all along [the Karuk] were trying to establish a historical right to their spiritual places. So that was tough, because it wasn’t their land. It was National Forest land under National Forest policies, not Karuk policies. So it was very hard to know how far you go.”

– Barbara Holder, retired Forest Supervisor, Klamath National Forest

The Ti Bar Demonstration Project was a remarkable and difficult shift in forest management and state-Indigenous relations, which evolved out of crisis moments and coercive forces (Wood and Welcker 2008:401) in U.S. forest management history (see Diver *et al*. 2010). In the early 1990s, following years of intensive industrial logging, the Forest Service was struggling to respond to the northern spotted owl listing under the U.S. Endangered Species Act (Table 1). In 1994, the Northwest Forest Plan (NWFP) ushered in new concepts of “ecosystem management,” which prompted the Forest Service to hire its first staff ecologists.

**Table 1:** Timeline of events related to the Ti Bar Demonstration Project

1979 – U.S. government confirms the Karuk Tribe’s status as a federally recognized tribe	
1983/84 – Karuk Tribe protests logging on sacred Offield Mountain	
1987 – Large wildfires occur across California, and in the mid-Klamath	
1988 – Karuk fisheries program begins as a precursor to the Karuk Department of Natural Resources	
1990 – Endangered Species Act (ESA) listing occurs for the northern spotted owl (threatened)	
1993 – Karuk Tribe is invited to join Klamath National Forests (KNF) Land and Resource Management Plan (LRMP) planning team	
1994 – Northwest Forest Plan is adopted	
1996 – Forest Service hires ecologist Jon Martin as the Ukonom and Orleans District Ranger	
1996 – Interagency Agreement between the USDI Bureau of Indian Affairs and USDA Forest Service, Klamath National Forest, October 4, 1996 is signed, authorizing the Ti Bar Demo project	
1997 – Karuk Tribe begins Ti Bar Demo planning	
1998 – Karuk Tribe completes Ti Bar mission and treatment proposals, which are adopted by the Forest Service	
1998 – Forest Supervisor Barbara Holder (KNF) retires; Forest Supervisor Martha Kettelle (SRNF) is transferred shortly afterwards, and her replacement does not support the Ti Bar project	
1999 – Tribal crews complete Ti Bar Demo willow treatment and forest thinning/pile burning	
1999 – Orleans and Ukonom District Ranger Jon Martin departs for D.C.	
2000 – The remaining Ti Bar treatments are not done; the planned underburn does not occur	
2005 – Karuk Environmental Management Practices Demonstration Area (KEMPDA) concept paper is signed to continue Ti Bar Demo, but does not result in any project activities	

The new forest policy shut down much of the existing timber harvest on public lands in the Pacific Northwest region, which contributed to a severe economic downturn for timber-dependent communities. One Forest Service representative commented on NWFP impacts to forestry in Klamath National Forest, “[We] went to harvesting 50 million board feet per year under the Plan. Before, it was five times that—at 250 million board feet per year.” Around this same time, the Forest Service was also dealing with large wildfires across California, Oregon, and Washington (Biswell 1989), including an increased number of large wildfires in the Klamath Mountains (Salmon River Restoration Council).

#### Initiating Collaboration Through Crisis

These forest policy changes were preceded by a growing tribal sovereignty movement in the Klamath Basin. For example, in the mid 1980s, Karuk tribal members and their allies staged direct action protests to stop a helicopter logging sale in cultural areas on Offield Mountain, a sacred place for Karuk people. It was this *de facto* assertion of tribal authority and associated press coverage that initially compelled the Forest Service to begin consulting with the Karuk Tribe. Karuk DNR director and ceremonial leader Leaf Hillman shared his perspective on Karuk-Forest Service relations, “You get to the point where people respect you out of fear. Not because it is the right thing to do.”

Following the Offield Mountain conflict, Karuk-Forest Service relations were at a low point. In the early 1990s, representatives from the Tribe and the agency began holding monthly meetings, often conflict ridden. During these discussions, agency staff offered to appoint a tribal member to the Forest Service Interdisciplinary Team that was developing a new Land and Resource Management Plan (LRMP) for the Klamath National Forest. Hillman referred to the offer as “one of the bones that they tossed out” because the opportunity to provide input at the final planning stage seemed minimal, but he volunteered for the job anyway.

Through the planning process, tribal leaders successfully lobbied the Forest Service to address several Karuk cultural resource management concerns. With significant pressure from Karuk Tribal Council, the 1995 Klamath Forest LRMP established the “Management Area 8” land use designation for Cultural Management Areas (CMAs), including primary Karuk ceremonial sites and the surrounding landscape. These areas require a signed Memorandum of Understanding between the Forest Service with the Karuk Tribe to support management activities that are “consistent with their [the tribe’s] custom and culture” (USDA Forest Service 1995). Before engaging in joint management at highly sensitive World Renewal sites, Karuk tribal leaders proposed developing a demonstration project at the Ti Bar area—not a primary ceremonial area, but still an important place for Karuk cultural use.

This initiative provides a first example of how the Karuk Tribe located a strategic pivot point within existing state planning processes. Despite limited tribal participation in the planning process, the Karuk Tribe leveraged the Forest Service LRMP process to establish formal cultural management areas. This strategy followed the agency’s existing policy approach of zoning for different types of land use, but it introduced a drastically different governance arrangement by requiring mutual agreement between tribal and agency representatives before management activities could take place. In this case, it was crisis conditions in forest management and tribal activism that compelled the Forest Service to adopt the LRMP policy change, which set the stage for the Ti Bar Demonstration Project co-management experiment.

### Transformational Moments: Experiments in Post-colonial Forestry

“My great grandmother told me a lot when I was young. . . . How she got telling me about fire was—I guess I was about 4 years old—and I was playing with matches by the stove. And she caught me. She told me if I was going to be playing with fire, then I ought to be doing something good with it. She took me outside, and we started burning hazel. By the time I was 8 years old, I was burning off big chunks of blackberries just by myself, even when no one else was home. Keeping control of it without any water or anything. It’s all about the timing and the conditions. And the way you light it. You can move it around and make it take its own energy out of itself. It’s kind of interesting. So it’s something that I just did, from 4 years old and on.”

– Bill Tripp, Karuk tribal member

The Ti Bar Demonstration Project was one of the first efforts to bridge the conflict-ridden relationship between tribal and agency managers in the mid-Klamath. In 1999, tribal teams working at Ti Bar completed several eco-cultural restoration projects to enhance cultural resources on federal forestlands. These included a willow stand treatment and a forest thinning treatment with pile burning over 189 acres. What follows highlights some the post-colonial planning moments that were realized through Ti Bar Demo co-management (Table 2).

**Table 2:** Key steps for the Ti Bar Demonstration Project

1) Strengthening Forest Service-Karuk relationships, demonstrating political will from the agency	
2) Identifying funding and overarching project goals	
3) Generating a “pivot point” through existing policy: Interdisciplinary Team co-leads	
4) Tribal planning for Karuk eco-cultural restoration	
5) Implementing prescriptions with tribal crews	
6) Project implosion: losing supportive agency leaders	

#### Achieving Joint Management

As a first step to creating Ti Bar Demo, Forest Service leaders strengthened relationships with tribal managers. The new ranger for the Ukonom and Orleans Forest Districts, Jon Martin, was an ecologist by training and played a key role in demonstrating genuine political will towards Forest Service-Karuk collaboration. For example, Martin organized informal events, such as a river float trip, that brought together tribal and agency leaders. Forest Supervisors had hired Martin to help the agency understand how to fulfill its new mandate for performing “ecosystem management.” Given his interests in ecological relationships, Martin was uniquely positioned to appreciate Karuk eco-cultural restoration concepts. Martin educated himself about Karuk culture by attending ceremonies and talking to tribal members about how they did things. Karuk tribal member and cultural biologist Ron Reed described Martin as someone who “hung around long enough to understand what we were talking about.”

The second step was generating funding for a co-management project, which would support restoration planning and implementation by Karuk land managers. Working with Forest Service allies, the Karuk Tribe identified a funding source to cover the real cost of a mid-Klamath prescribed burning project, since site preparation required extensive mechanical thinning. Karuk managers then worked with the Forest Service and the BIA to develop a legal agreement authorizing project goals and funding distributions to the Tribe.

The Karuk Tribe spent several months developing its priorities, including Karuk eco-cultural restoration principles. The term eco-cultural restoration refers to restoring dynamic ecosystems and human cultures together as interconnected processes. In the case of Ti Bar Demo, applying Karuk eco-cultural resource management meant using prescribed fire and other restoration strategies to actively manage and enhance understory plants and wildlife that are most important for Karuk subsistence and ceremonial uses.

A third and central element to Ti Bar Demo was identifying the Interdisciplinary Team (ID Team) as the primary mechanism for supporting co-management. District Ranger Martin identified ID Team co-leaders, or “co-leads,” from the Tribe and the Forest Service. Despite the agency’s history of excluding Karuk people from decision-making, the ID Team was an established policy framework authorized by the National Environmental Policy Act of 1990 (NEPA) that enabled agency managers to work with tribal managers as a new kind of expert.

The co-lead structure was a very different approach from what Forest Service staff were accustomed to, and not all were comfortable with it. The Forest Service has typically used the ID Team format to bring together agency experts for environmental assessments, and some staff members resisted the idea of appointing a tribal representative as a full ID Team member. They questioned whether the Tribe had the professional expertise to conduct an environmental assessment. Still, the co-lead arrangement moved forward under the District Ranger’s authority.

The significance of the co-lead arrangement was that it enabled Karuk tribal crews to take on a primary role with implementing their own restoration projects. By being ‘‘on the ground,’’ Karuk land managers had the flexibility to conduct eco-cultural restoration treatments in a culturally sensitive and adaptive manner that responded to site-specific conditions. For example, tribal crews could strategically locate brush piles for burning to avoid patches of tan oak (matsutake) mushrooms, an important traditional food that is sensitive to fire. Tribal member and eco-cultural restoration specialist Bill Tripp expanded on the importance of having tribal crews,

“While we were out there, we were cutting hazel. We were doing different things like that. We didn’t say in the prescription that we were going to cut 50 hazel patches per acre. We just said that we were going to cut, limb trees, and pile and burn brush less than a certain diameter. But just that kind of prescription was enabling [for] us. We were out there doing it. We were able to do … [what] … we needed to do to enhance the resources. Though not specified in the prescription, we were able to reduce fuels while leaving species such as mature yew and dogwood that are typically smaller in diameter. So in that sense, we couldn’t meet the prescription in its entirety. But we were able to make on-the-ground decisions that reduced potential [catastrophic] fire effects as needed.”

In this way, tribal managers were trying to find a middle ground between formal prescriptions and Karuk management principles. Not all of the crew’s activities were officially approved by the Forest Service and this was part of what it meant to move towards a post-colonial planning model. According to Bill Tripp:

“So we let a pile get away from us at lunch. And then put it out after lunch. And it burned out a nice little area about the size of this room, like four hundred square feet. That was a little bit outside the scope of NEPA [the National Environmental Policy Act], but we did it. It needed to be done. It had been a long time. We shouldn’t have to write NEPA to be an Indigenous people. So I’m not afraid to say that we went a little bit outside the box, because that’s exercising our sovereignty. We wouldn’t be exercising our sovereignty if we didn’t stretch those limits on occasion.”

The ID Team strategy is another example of a pivot point that enabled the Karuk Tribe and its allies to work within existing rule systems to shift standard policy. The ID team framework offered Karuk managers a preliminary “fit” with agency policy and procedures. Martin’s co-lead structure then pushed back on the existing policy frameworks to facilitate a meaningful decision-making role for tribal members, regardless of resistance from agency staff.

These elements of Ti Bar Demo co-management all contributed to a post-colonial forestry approach that increased power sharing between the Karuk Tribe and the Forest Service. Although Karuk-Forest Service working relationships were far from perfect, the ID Teams enabled Karuk managers to play a meaningful role in forest management. By creating additional legitimacy for Karuk perspectives, the ID Teams facilitated important social learning opportunities, which led to improved understanding among co-managers over time. Unfortunately, as events unfolded, the Karuk Tribe and its allies were unable to complete all of the planned eco-cultural restoration treatments at Ti Bar.

#### Project Implosion

The breakdown of Ti Bar Demo began with a turnover in Forest Service leadership in 1998-1999. Without support from Forest Service leaders or agency staff, remaining restoration treatments were left undone. Around the same time that the first Ti Bar treatments were initiated, key project supporters Barbara Holder and Martha Ketelle, the respective Forest Supervisors for the Klamath and Six Rivers National Forests, left their positions. The new Six Rivers Forest Supervisor was not supportive of District Ranger Martin’s approach on Ti Bar Demo or other collaborations with the Tribe.^.^ 1 As a result, Martin left the Klamath in the fall of 1999. Martin recalled the course of events:

“When I first got to the ranger job, I had a couple years of supportive Forest Supervisors. . . Midway through, I got a new boss with a 180-degrees different philosophy than either of the previous supervisors. The new boss was very uncomfortable with shared decision-making and collaboration, especially with the Karuk Tribe. This was what I would call an old school philosophy . . . [that] reflected the attitude, ‘this is my ranch and I’m running it’ . . . And the staff was very much in the [same] mode with my new boss. [In their view] we were the ones to propose the projects, as the paid professionals. . . . I saw the writing on the wall and left for D.C.”

After Martin’s departure, Karuk tribal managers learned that remaining restoration treatments, including the planned underburn, would not move forward, and the project ID Team fell apart. This occurred even though the Forest Service had listed the planned underburn on its official program of work. Several years later, the Tribe attempted to revive the Ti Bar Demonstration project without success.

#### Understanding Co-management as a Catalyst

Understanding Ti Bar Demo impacts in their entirety requires reframing co-management as a catalyst. As a short-lived initiative, Ti Bar Demo was only a temporary experiment in post-colonial forestry practices. The abandonment of Ti Bar Demo exposed the weaknesses of the formal agreement authorizing the project, which was highly contingent upon the awareness, acceptance, and political will of individual agency leaders. In retrospect, the project required additional accountability measures to ensure that the agreement would be upheld, irrespective of changing government priorities or staff turnover. A greater level of legal accountability or *de jure* rights are needed if co-management arrangements, such as the Ti Bar Demo, are to become more than a temporary space for negotiating knowledge and authority between parties.

However, tribal managers ultimately view the project as a success. This was the first time that Forest Service leaders had formally recognized the rights and ability of tribal managers to manage cultural resources within federal forests. The District Ranger worked hard to support tribal managers by identifying a pivot point within existing policy that enabled a co-management approach. Restoration treatments by tribal crews improved tribal access to cultural resources by enhancing local hazel and willow patches for basket making. Irrespective of the Forest Service’s jurisdiction over federal forests, Ti Bar restoration treatments supported many Karuk goals, including the Tribe’s inherent responsibility under Karuk World Renewal philosophy for stewarding the diversity of wildlife, plants, and non-human entities that make up the mid-Klamath landscape. Facilitating tribal capacity building, the project generated funding for the nascent Karuk DNR and created much-needed jobs for tribal members. Furthermore, by increasing the legitimacy of tribal management institutions, the project supported the Karuk Tribe in building alliances that extend far beyond the Forest Service. Working through these alliances has repositioned Karuk managers as thought leaders on prescribed fire at the regional and national level, thereby increasing the influence of Karuk management institutions across a network of nested institutions.

The Ti Bar case confirms that co-management is not a panacea (e.g., Borrini-Feyerabend *et al*. 2004) or an end point. Rather, it is an interim strategy that can support broader Indigenous self-determination initiatives, particularly when communities have the capacity to negotiate with state agencies. When it is included within existing policy frameworks, co-management can itself function as a pivot point, providing communities with a tool for negotiating power-sharing despite agency structures and norms that disempower Indigenous communities, Thus, Ti Bar Demo can be best appreciated as a component of the Karuk Tribe’s long-term strategy for self-determination and environmental sustainability.

### Attending to Co-management Realities: Structural Barriers to Equity

“Twenty years ago, co-management was a term that we didn’t dare use . . . For Forest Service district rangers . . . co-management implied that authority was being given away. District Rangers didn't have the legal basis to give this [authority] away. However, at the time (and still today), there were ways to share in decision-making through collaboration and partnerships . . . as long as the decision-makers ‘up-the-line’ were supportive.”

– Jon Martin, former Ukonom and Orleans District Ranger

This case study speaks to the deeper challenges of equity that are raised in the co-management literature. Despite some of the positive moments with Ti Bar Demo, all interview respondents commented on the difficulties they experienced in attempting to realize joint decision-making between the Karuk Tribe and the Forest Service. Unpacking these difficulties reveals some of the structural barriers faced by co-managers and the forces of co-optation that are intrinsic to the co-management process.

1. *Sharing decision-making authority.* One barrier to co-management was a lack of agency understanding of how different laws and policies intersect to authorize co-management. Some Forest Service staff voiced concerns about the potential illegality of giving away management authority vested in their agency by the federal government, e.g., through the U.S. Administrative Procedure Act. Some commented that they are unable to share authority with tribes due to the agency’s mandate to serve the broader public. Others were concerned that non-tribal stakeholders would accuse the Forest Service of being “unfair” or giving “special treatment” to tribes. Tribal members note that these viewpoints position the Karuk Tribe as one of many interest groups, not as a sovereign nation, a positioning that overlooks principles of U.S. federal Indian law.
  Tribal members and Forest Service staff had different views about who is entitled to management authority, and where this authority comes from. For example, tribal members found that many Forest Service leaders brought a sense of ownership over the landscape to their position, an attitude that offended many of them. As one tribal member commented,
  “The Forest Service is fairly new in the Tribe’s life in this area. The Tribe has been here much longer. . . and feels more of an attachment to the land than what they see as the transient Forest Service people coming and going. We’ve been through a lot of rangers here. But each ranger will seem to have this attitude that this is *their* district. I’ve even seen it come with rangers on their very first day they show up to work at the district. And it’s like, ‘Ok, this is your district? A lot of people have been here for a long time, long before you even thought about coming here.’ I don’t know that they’ve really thought about it or know that they are coming across as very disrespectful to the people that had already been there.”
2. *Acknowledging tribal expertise.* A second barrier was Forest Service assumptions about the nature of expertise, particularly the issue of whose knowledge counts in land management decisions. Even though important knowledge exchange occurred in the Ti Bar case, Western knowledge traditions privileging “scientific expertise” were still valued over Karuk knowledge. Instead of a collaborative learning opportunity, some tribal representatives saw the Ti Bar project as a process for learning how to disguise Indigenous knowledge within Western science formats.
  Part of the problem was a difference in learning systems. The Forest Service typically hires staff with technical training, obtained through higher education programs. In contrast, some Karuk tribal managers had not received their high school diploma. One Karuk DNR staffer reported that they taught themselves how to read and write when they began working for the Tribe. Tribal managers were selected for their jobs because they had a Karuk cultural perspective on land management, which they gained through experiential learning with knowledgeable elders and family. They learned the technical skills they needed on the job. Tribal member Ron Reed reflected on how this problem affected his experience as Ti Bar Demo co-lead,
  “They had their maps. They had their titles. They had their privileged education that provided that [agency] resource management perspective. And if you didn’t relate to them on their level of education, you didn’t register. I felt insignificant. I couldn’t chime in on their conversation because I developed an inferiority complex. I didn’t understand their catch phrases, acronyms, or policies. I found out I had better learn this information. But what jumps [out] at me also right now is that we need to make our own rules. We need to assert our own names, our own maps.”
3. *Negotiating knowledge and values.* Another barrier was the difference between federal agency and tribal laws. The problem was not so much that such differences exist. Rather, the issue was how that difference is accounted for given competing mandates (e.g., Hillman and Salter 1997). The Tribe is typically required to “meet or beat” federal laws and policies, while Karuk laws and land management principles are often overlooked. This practice places Karuk ways of knowing in a subordinate position and discourages equitable knowledge sharing.
  In its defense, Forest Service leaders participating in Ti Bar Demo supported ecosystem management, an approach that aligned with many Karuk values. In addition, the District Ranger helped champion Karuk management goals and facilitated meaningful co-management negotiations. Still, making the shift towards valuing non-merchantable cultural resources and foregoing potential profits was difficult for other Forest Service staff involved with the project. Ron Reed commented on this disconnect,
  “[The sentiment from the Forest Service was] ‘How are you going to get your money back if you are managing for acorn trees? . . . Over here, we’re managing a million dollars worth of timber. But over there, we’re not managing for anything. It’s a waste of time.’ So that’s what I learned about Traditional Knowledge. It wasn’t looked at the way it should have been.”
4. *Assumptions of objectivity.* Co-management barriers included Forest Service assumptions around “objectivity” and “professionalism”—characteristics that some Forest Service managers ascribed exclusively to agency personnel. As “paid professionals,” a number of Forest Service staff felt that they should be the ones making decisions in Ti Bar process. In contrast to “professional” agency staff, tribal members were viewed as being less qualified.
  As a related problem, multiple Forest Service staffers who engaged with the Tribe reported being accused of partisanship. A non-tribal person working for the agency said they were referred to as an “Indian sympathizer” and felt marginalized at work. One tribal member working for the Forest Service recalled another staff member referring to their presence at an internal planning meeting as “a conflict of interest” for the agency. A different tribal member reported sharing cultural perspectives when they first started their Forest Service job, but they soon stopped after learning that this was viewed negatively by their peers.
  Such attitudes about bias—that involving tribal members in agency decision-making would corrupt what would otherwise be a fair and balanced process—are highly problematic in part because the theory of an impartial or interest-free management agency is a poor representation of reality. Historically, the Forest Service has brought its own political interests into management decisions (e.g., Schiff 1962). And as argued by Harding (1995), all individuals bring their personal interests and cultural background into decision-making processes, and it is precisely the recognition of multiple “situated” perspectives that leads to more effective decisions.
  While acknowledging the high degree of heterogeneity within and among Forest Districts, and recognizing that institutional norms change over time, these comments suggest a history of “othering” Karuk perspectives within the agency. Many Karuk tribal members see themselves a having a distinct perspective on land management, which could be useful for both tribal members and the general public. One individual described the agency’s marginalization of tribal members as a missed opportunity,
  “Okay, here’s an opportunity. I work for you, [the Forest Service]. I know what I need to say to you to communicate with you in the structure that makes sense for the Forest Service. And then I also know how to talk to the Tribe because I grew up as a tribal member. So you kind of have this bridge. It was never received that way. . . . the Forest Service felt that because [you are] a tribal member, that your loyalties lie there. Your loyalties don’t lie with us, the Forest Service. . . . So because I felt that the Forest Service didn’t want me like that, I didn’t push it. I didn’t make a nuisance of myself.”
5. *Accounting for colonial legacies.* Another barrier was the Forest Service’s history with facilitating the displacement of Karuk people from their homelands. Tribal members readily recall Forest Service policies at the turn of the century that criminalized Native American burning and established federal ownership over the majority of Karuk territory. More recent Forest Service decisions evicting “squatters” from old mining settlements have reproduced these colonial histories for the Karuk. Tribal members are quick to explain that settling on old mining claims was one of the few ways that Karuk families could continue living in traditional family areas. This was due, in part, to the discriminatory practices of government officials approving tribal allotments, i.e., parcels of land deeded to individual Native American families.
  For this reason, tribal members are often highly distrustful towards Forest Service employees. This distrust extends to other tribal members working for the agency. For example, one tribal member in the Forest Service reported that certain family members view them as a “traitor.” In the Ti Bar case, District Ranger Jon Martin found that it was extremely difficult to build a working relationship with tribal members, despite his willingness to champion Karuk land management goals. As Martin explained,
  “It was like driving down the highway and hitting a brick wall. Because [many] people insisted on blaming me for everything that had happened in the last one hundred years. It was not really about me personally. It was about my role in the agency. But that was rather painful until I figured it out.”

Considering the barriers discussed above provides additional perspective on the immense challenges faced by Ti Bar Demo supporters. Even with its positive outcomes, Ti Bar Demo did not overcome existing power hierarchies or deeper areas of difference among co-managers. In asserting their interests with the Forest Service, Karuk tribal managers often encountered institutional structures and norms that reinforced colonial histories. This analysis of Karuk-Forest Service relations suggests that greater recognition of ongoing colonial legacies and the embeddedness of Klamath forests in non-Western cultural traditions is needed if the agency wishes to pursue opportunities for healing and reconciliation (e.g., Middleton 2010).

## Conclusion

The Ti Bar Demonstration Project represents one of the Karuk Tribe’s first attempts to co-manage restoration projects on federal forestlands. Although Ti Bar Demo affirms many co-management critiques, this case also supports previous research findings that incremental change is valuable (Borrini-Feyerabend *et al*. 2004). Ti Bar Demo outcomes are positioned in between the extremes of transformation and co-optation often presented in the co-management literature. In this case, the Karuk Tribe engaged with existing state policy frameworks along with their limitations—while simultaneously pushing back to change those frameworks and address the Tribe’s self-determination goals. The pivot point concept emphasizes the importance of identifying leverage points within existing policy as a starting point for negotiations that can catalyze policy change.

Cases like Ti Bar Demo demonstrate how natural resource management decisions are layered on top of colonial legacies, which can lead to significant environmental justice issues. Too often, tribal communities that have experienced disproportionate impacts from extractive resource management must allocate significant resources to engaging with institutional structures that do not account for cultural diversity. Many tribal managers would prefer to start fresh and create their own Indigenous resource management institutions—as opposed to working within the constraints of existing state institutions and the relations of power and privilege that are embedded within them. But creating Indigenous resource management initiatives in a multi-jurisdictional context requires time and resources for capacity building, both for tribes and state agencies. As an interim strategy, leveraging tools like co-management can help build tribal capacity in resource management decisions, when this approach fits with an Indigenous community’s broader self-determination goals. Thus, co-management can be understood as one step towards increasing equity in natural resource management and realizing a new paradigm of post-colonial forestry.

## Footnotes

1 Because of persisting sensitivities around agency-tribal relations, I have identified the Forest Service employees involved by position and not by name.
